# Student nurse‐educators' construction of teacher identity from a self‐evaluation perspective: A quantitative case study

**DOI:** 10.1002/nop2.75

**Published:** 2017-02-23

**Authors:** Ferdinand C. Mukumbang, Leka Marcel Alindekane

**Affiliations:** ^1^ School of Public HealthFaculty of Community and Health SciencesUniversity of the Western CapeBellvilleSouth Africa; ^2^ School of NursingUniversity of the Western CapeBellvilleSouth Africa

**Keywords:** competencies, diadactic expert, nursing education, pedagogy expert, student nurse‐educator, subject matter expert, teacher identity, teaching and learning, teaching practice

## Abstract

**Aim:**

The aim of this study was to explore the teacher identity formation dynamics of student nurse‐educators about the subject matter, pedagogy and didactics.

**Design:**

A case study using descriptive quantitative design was employed.

**Methods:**

Using a cross‐sectional approach, data were collected in 2014 using a self‐administered questionnaire. Participants were asked to self‐evaluate their teaching competencies on the nursing subject matter, pedagogical expertise and didactical expertise. Using descriptive analysis we determined the central tendencies of the constructs.

**Results:**

The descriptive analysis revealed a very small variance (0.0011) and standard deviation (0.04) among the means of the three constructs, which indicates a fair balance in the contribution of the subject matter, pedagogy and didactics towards teacher identity formation. Nursing student‐educators can achieve a balanced combination of subject matter expert, pedagogical expert and didactical expert combination during the formation of their teacher identity. This could be indicative of how effective the training programme is in helping the students achieve a balanced teacher identity.

## 1 Introduction

The quality of education produced by educational institutions depends on the quality of the teachers they possess as students' learning is directly related to their interaction with the teachers (Department of Higher Education and Training, 2014). Effective teaching, therefore, depends on the knowledge, skills and commitment that the teacher brings to the teaching‐learning process. In other words, what the teacher knows and can do makes the crucial difference in what the teacher can accomplish (Feiman‐Nemser, 2012).

Nursing education programmes to train nursing educators (teachers) are aimed at providing competencies that may be achieved by student nurse‐educators—nurses receiving teacher training to become nursing educators—on completion of the training programme. These student nurse‐educators are expected to focus on grounding themselves in the subject matter acquiring the instructional tools of teaching and classroom management. This requires that the student nurse‐educators should acquire competencies of the knowledge of subject matter, knowledge of teaching strategies, and tools to create and sustain an effective learning environment. Apart from achieving these competencies, they are expected to start formulating a professional teacher identity. It is assumed that if the student nurse‐educator acquires the above competencies (subject matter, pedagogy and didactics), then developing a teacher identity—how teacher candidates negotiate personalized understandings of themselves in programmatic context—becomes possible (Schepens, Aelterman, & Vlerick, 2009). It must be noted, nevertheless, that forming a professional identity is a continuous process related to one's interpretation and reinterpretation of their professional experiences and contextual factors (Rusa, Tom, Rebega, & Apostol, 2013).

Studies have shown interest in the educational process between student nurse‐educators and students in the classroom. These studies focus mainly on how the nursing students (the ones being taught to become nurses) construct their nursing professional identity during their clinical placements. However, interest in studying nursing professionals in their role as teachers, academics and university researchers has been scarce (Aguayo‐González & Monereo‐Font, 2012). In this study, we look at the dynamics around formulating a professional teacher identity by student nurse‐educators during their practicum period. We focus, in particular, on how they perceive their roles as a subject matter expert, a pedagogical expert and a didactical expert to explore how well they formulate their professional teacher identity. It was deemed important to study the formation of student nurse‐educators' teacher identity during teaching practice because they are expected to fully assume and identify with being nurse educators.

### 1.1 Background

#### 1.1.1 Teacher identity

Many educators have located teacher preparation inside apprenticeship models of learning to teach. Aligned with these perspectives and practices, teacher identity draws attention to the holistic, dynamic and situated nature of teacher development (Ronfeldt, 2012). In recent decades, professional teacher identity has received greater attention (Akkerman & Meijer, 2011; Beauchamp & Thomas, 2009). There is an assumption that learning to teach is crucial to the professional teacher identity development (Olsen, 2010; Özmen, 2010). According to Alsup (2006) “identity development for a teacher is complex, and that establishing a rich multi‐faceted identity requires the acceptance of ambiguity, multiple subjectivities, shifting contexts, and uncomfortable tension between ideological perspectives” (p.16). Acknowledging and dealing with the fundamental pressures of becoming a teacher requires attention to the ways in which individuals move within and between diverse spaces, especially through the contexts of schools and university in relation to the curricula (Beijaard et al. 2004).

How student nurse‐educators make sense of their teacher identity evolves out of the developmental capacities of the “self”. Robinson (2012) stated that “the conception of self suggests the way student nurse‐educators make sense of their experiences” (p. 89). He further reported that while developing the teacher identity, there is an emergence of self‐concept, a consistent notion of me, and an enduring set of dispositions. The success of this developmental stage stems from the fact that one is a “project for oneself”. The student nurse‐educator at this developmental stage is likely to conceive of the teacher role as a means to fulfilling his/her own purposes.

The preparation programme, especially the practicum period deliberately and inadvertently reinforces the development of different kinds of teaching identities as they emphasize various aspects of what it means to be a teacher. This places the nursing teacher candidates in different environments, where they will experience certain kinds of norms which they are expected to adopt (Lim, 2011). Although this aspect of preparation is not always explicitly considered, it plays a pertinent role in shaping student nurse‐educators' identity. This is realized as students develop their identity as members of the social group they work in, which reshapes their own views and what is understood about others.

Research on social identity argues that through childhood and into adulthood, people move through a variety of stages in making sense of their own social identity and cultures and those of others (Jurasaite‐Harbison & Rex, 2010). This process of social identity development influences how humans see their role in confronting social and institutional barriers to equity. The process of developing a teacher identity can be facilitated by teacher education if teacher educator candidates understand the different realities during the construction of teacher identity.

Depending on the educational environment in which student nurse‐educators develop, their self' concepts may be undermined, when they are confronted with different views of the world. In addition, the positive or negative role models in their past may shape their professional self‐image of nurse educators. Behaviour is, therefore, a function of self‐concept, making it an essential aspect of teaching and learning to teach (Day & Gu, 2010). A distinction should be made here between the teacher as a personal‐self and professional‐self to avoid the confusion. The first entails an organized summary of information, rooted in observable facts concerning oneself, which includes such aspects as traits of character, values, social roles, interests, etc. While the latter encompasses the first, it also emphasizes reference to one's self‐professional functioning. However, this distinction should not separate the individual nurse educator (teacher) from his/her professional performance.

#### 1.1.2 Teaching Practice

Teaching practice is the practical application of teaching methods, teaching strategies, teaching principles, and teaching techniques to enhance teaching skills in to be teachers. It involves practical training and exercises using different activities of daily school life (Wanekezi, Okoli, & Mezieobi, 2011). The term teaching practice has three major connotations: the practicing of teaching skills and acquisition of the role of a teacher; the whole range of experiences that students go through in schools; and the practical aspects of the course as distinct from theoretical studies (Lim & Morris, 2009). Student teaching practice can be conceptualized as space for prospective teachers to explore and continue to renegotiate their identity in relation to the subject to be taught.

Very often during teaching practice, much focus is directed to the delivery of lessons, and the feedback from the supervisors deals with issues of knowledge and skills in teaching. Consequently, campus‐based course teaching practice should be aligned with the theoretical and evidence‐based teaching procedures taught in the methods course to foster a meaningful teaching experience (Zeichner, 2010). The campus‐based course plays an important role in the development of student nurse‐educators by placing emphasis on the quality of their teaching practice. The programme design should be comprehensive enough to cover different teaching strategies that could develop a concrete theoretical basis of their teaching. The subject matter courses should equip them with the substantial subject knowledge to comprehend or facilitate the curriculum. The pedagogy courses should equip them with substantial pedagogical content knowledge so that they are confident in their teaching practice (Godino, 2009).

Researchers have identified the unequal link between theory and practice as one of the basic problems in teacher preparation programmes. They propose a paradigm shift from one theory to practice to a paradigm of integrating theory and practice (Krull, Oras, & Pikksaar, 2010). Therefore, teaching practice should serve as an important element in which theory and practice are integrated to improve quality in initial teacher education. The combination of theory and practice emphasizes that teaching practice should provide an opportunity for the acquisition of the subject matter, pedagogical and didactical knowledge in achieving the professional tenure and development of teacher identity. Therefore, during the practicum period of student nurse‐educators, they are expected to develop themselves as the subject matter experts, pedagogical experts and didactical knowledge experts as they engage in real‐life teaching experiences.

The practicum period for the researched institution is 6 months teaching exercise whereby student nurse‐educators are required to give at least 10 classroom lessons on various nursing content and five nursing practical skills to undergraduate nursing students in real‐life situations. They are expected to prepare a lesson plan, design an evaluation tool for the session and deliver each of the lessons as part of their development process. During the teaching sessions, they are expected to use a variety of teaching methods (problem‐based learning, case based learning, lecture etc.) and tools to deliver their lessons. During these lessons, they are evaluated by their educators (supervisors), the students they are teaching and the teacher who normally takes the lessons. At the end of the practicum period, the student nurse‐educators are required to compile a portfolio for their teaching practicum.

During these teaching sessions, the student nurse‐educators are expected to play the role of seasoned nurse‐educators because of the responsibilities they have to transmit knowledge to the students they are teaching while at the same time, they are being judged on their teaching skills by their educators (student role). This leaves the student nurse‐educator with a dual “identity”. The identity of being a student and the identity of being an educator, both to be displayed at the same time. Managing these two identities becomes a challenge to many of this student nurse‐educators as one of these identities usually overshadows the other during the teaching practice sessions. This state is described by Pillen, Den Brok and Beijaard (2013) as professional identity tensions. According to the authors, professional identity tensions are usually experienced when the student nurse‐educator fails to reconcile their personal and professional sides of becoming or being a teacher.

With the ultimate goal of the practicum period being the acquisition of competencies related to subject matter, pedagogical and didactical knowledge, our focus in this study was to explore to what extent the overall study cohort formulated a pooled professional teacher identity. In other words, how well as a group, could they balance the subject matter expertise, pedagogical expertise and didactical knowledge expertise towards developing their teacher identity. The aim of this was to give the investigators an idea of the success of the practicum programme in developing the professional teacher identity.

#### 1.1.3 Theoretical framework

**Figure 1:**
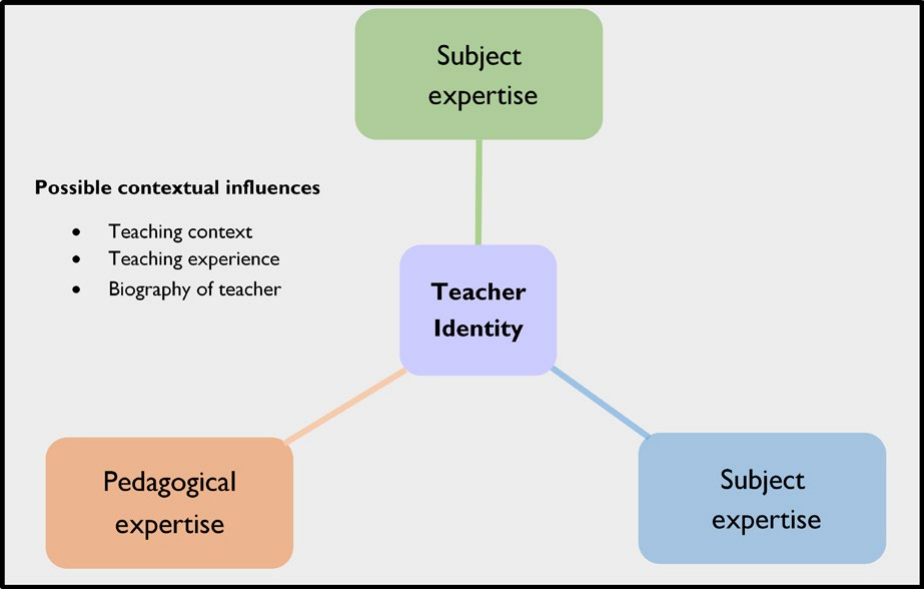
Conceptual framework adapted from Beijaard et al. (2000a,b)

There are multiple theoretical frames that have been previously developed to explain the development of teacher identity. We adopted the framework proposed by Beijaard, Verloop, and Vermunt (2000a) and Beijaard, Meijer, and Verloop (2000b) to explore how nursing student teachers assume and construct the teacher identity. This framework conceptualizes the identity of teachers with respect to their strengths in subject matter, pedagogy and didactics as Beijaard et al. (2000a,b) believe that expertise in subject matter, didactics and pedagogy is central to the development of teacher identity. The investigators used this framework (Figure 1) to explore the formation process of teacher identity.

The teacher as a subject matter expert in the Beijaard model refers to possessing and understanding the subjects being taught, having knowledge of designing the learning process and the ability to transform subject matter into content. A good grasp of the theories and concepts pertaining to a specific subject matter plays a fundamental role in the development of teacher identity. A competent teacher has a thorough knowledge of the subject matter. The teacher who has command over subject matter can provide more information to the students in the classroom (Ruohotie‐Lyhty, 2011). Didactics expertise is the knowledge necessary for designing the learning process, and pedagogical expertise is knowledge related to the understanding of human thought, behaviour and communication (Lindström, 2011). This entails the relationships, values and the moral as well as the emotional aspects of development. The teacher as a pedagogical expert refers to the ability to transform the subject matter into content such that it can be transmitted to the students.

The concepts of subject matter, pedagogy and didactics are not separated from each other, rather, they all influence an individual's teacher identity (Figure 1). According to Beijaard et al. (2000a,b), teacher's professional identity can be described in terms of the teacher as a subject matter expert, the teacher as a pedagogical expert and the teacher as a didactical expert. Using this framework, the authors, worked on the assumption that possessing a good grasp of ability the subject matter, mastery in pedagogy and good didactic abilities are essential for constructing a fair teacher identity.

### 1.2 Objective

To explore the teacher identity formation dynamics of student nurse‐educators based on how they saw themselves as subject matter experts, didactical expert, and pedagogical experts during their teaching practice. The goal is to obtain a pooled score of the cohort to gauge how well or not they have formulated their professional teacher identity as a cohort rather than individually. Thus, the goal is to describe the teacher identity value of a sample of student nurse‐educators (cohort) in a nursing school programme.

## 2 Methods

We employed a cross‐sectional descriptive quantitative research design. For this study, a quantitative research paradigm was used to ascertain the perceptions of student nurse‐educators regarding their teacher identity (Shields & Rangarjan, 2013). A descriptive design was used because the investigator intends to observe, count, delineate and classify the perceptions of student nurse‐educators regarding their teacher identity based on their perceptions of their personal knowledge (Polit & Beck, 2010). An evaluation questionnaire was used to obtain information in a standardized manner. Student nurse‐educators, registered for the Master's in Nursing Education programme, from 2011 to 2013 at the School of Nursing in the institution of interest, defined the study population. Because only a total of 50 students nurse‐educators were registered between 2011 – 2013 (cohort), they were all included in the study. Of the 50 participants who made the sample frame, only 36 participated in the study. Ten were not available to respond to the questionnaires and the other four were used to pilot the questionnaire. These were not included in the study population. The following were considered as criteria for inclusion in the study: (1) All student nurse‐educators, registered in Masters in Nursing Education programme from 2011 to 2013 of the School of Nursing at the University of the Western Cape. (2) All student nurse‐educators who completed their theoretical course and teaching practice. (3) All student nurse‐educators who took part in the teaching practice from the year 2011 to 2013.

The questionnaire explored the three constructs (subject matter, pedagogy and didactics). The first section covered information on gender, age and education level. The second section included 15 items, five for each factor influencing the formation of teacher identity (context, experience and biography). The student nurse‐educators were asked to what extent they agreed with the items on a four‐point scale (ranging from 1: strongly disagree, to 4: strongly agree). In the third section of the questionnaire, the participants were asked to represent their teacher identity by awarding a total of five points on items constituting the three aspects of their identity (subject matter, pedagogy and didactics).

Questions that were posed required them to indicate how they rated themselves during their teaching practice on the subject matter, pedagogy and didactics. The categories were scaled so that the sum of the three variables (subject matter, pedagogical and didactic expertise) for each respondent equalled the teacher identity. To achieve this objective, a scale with five‐point response categories was constructed with an equal number of positive and negative labels anchored by opposite poles, and with mid‐points. It was used to measure the teacher identity through the level of knowledge of, expertise perceived in subject matter, pedagogical and didactic as rated by respondents.

### 2.1 Validity of data collection tool

The face validity of the questionnaire was gained through subjective judgements by means of lecturers who are experts in the concerned field. Because we sought to ascertain the perceptions of student nurse‐educators' teacher identity from a personal knowledge perception, we ensured that the questionnaire was representative of the three domains (subject matter, pedagogy and didactics expertise) that cover teacher identity. Selecting and defining the concept, specifying the intended claim, identifying the target population, and drafting the target product profile were the initial steps in assuring and documenting content validity.

The determination of teacher identity variables for this study entailed a content validity process. The procedure for establishing content validity was an adaptation of the process used for an existing instrument. Two experts in nursing education and one in research methods examined the content of the items and, based on specified definitions of the variables subject matter, pedagogy and didactics expertise, evaluated whether or not the items were consistent with the framework used (LoBiondo‐Wood & Haber, 2010). Although the perception of student nurse‐educators cannot be measured with the same precision as pure scientific variables, it is generally accepted in the social sciences that self‐reported data can be regarded as interval variable (Kerby, 2014; Pallant, 2011). Such authorities maintain that treating self‐reported scales as interval variables is most realistic if the scales have at least four possible values and the variable distribution is “nearly normal” (Kerby, 2014, p.168). Given the number of items and the complexity of the concepts involved, we considered the reliabilities of the scales to be acceptable.

### 2.2 Analysis

A descriptive data analysis was conducted with the use of Statistical Package for Social Sciences (SPSS) 22. In the first step of the analysis, the investigators transformed the 5‐point Likert scale responses into ordinal data, where the responses were awarded a number starting from 1‐5. The mean, standard deviations, variance and skewness of the various constructs were computed. The second step involved using the means obtained from the student nurse‐educator's perception of the various constructs (subject matter, pedagogy and didactics) to calculate the total mean (mean‐item‐means), which hypothetically represents the teacher identity. We considered the mean value of each of the three constructs that form teacher identity as the value that the nurse student‐educators (participants) attributed to that construct on teacher identity based on their self‐evaluation. Next, we computed the mean value of the means (mean‐item‐mean) of the three constructs to form a summed mean that we considered representing the teacher identity value. To investigate the extent to which the various constructs contributed to the formation of the teacher identity, we considered the variance of the construct means, to obtain the volatility of the constructs from the mean item mean. The variance was calculated using the formula below:

$$\sigma^{2}=\frac{\sum {\left(X-\mu \right)}^{2}}{N}$$

where σ^2^ is the variance, *X* is the score, μ is the mean, *N* is the number of scores and Σ is the summation.

The standard deviation (mean item standard deviation) was also computed to add more value to the degree of variability between the means item mean.

### 2.3 Ethical considerations

The study received ethics clearance from the University of the Western Cape Senate and Higher Degree Ethics Committee. Permission to conduct the study was obtained in writing from the Director of the School of Nursing and Head of the Nursing Department of the researched institution.

The respondents were given an informed consent form regarding taking part in the study after they had each received a written explanation of the study. Consent to participate in the survey was obtained from the students by signing a research consent form. Respondents were assured of the confidentiality and anonymity of any information provided during data collection and the data analysis processes.

## 3 Results

### 3.1 Characteristics of study participants

A total of 36 student nurse‐educators participated in the study. Table 1 below describes the characteristics of the study participants.

**Table 1:** Characteristics of student nurse‐educators

Characteristics	*N*	Per cent
Age		
28–32	3	8.4
33–38	13	36
39–42	13	36
43–47	7	19.6
Gender		
Female	32	88.9
Male	4	11.1
Grade level		
First year	8	22.2
Second year	28	77.8

### 3.2 Descriptive statistics

The results obtained for the mean item mean, variance and mean item standard deviation are displayed in Table 2 below.

**Table 2:** Descriptive statistics

	*N*	Mean	*SD*	Variance	Skewness	
	Statistic	Statistic	Statistic	Statistic	Statistic	*SE*
Subject expertise average	36	3.49	.333	.111	.215	.393
Pedagogical expertise average	36	3.56	.317	.101	.704	.393
Didactical Expertise average	36	3.56	.296	.088	.214	.393
Valid N (listwise)	36					

A mean‐item‐means value of 3.54 was obtained, which was used to calculate the variance. A variance value of zero indicates that all the values within a set of numbers are identical. This is the situation of a hypothetically perfect teacher identity, where the values of the three constructs that form the teacher identity are given equal weights or the student nurse‐educators feel comfortable in all three aspects equally. The variance .0011 was obtained. Normally, a large variance is indicative of how far the set is from the mean and each other while a small variance means that the set is close to the mean and each other. The variance value of .0011 is quite close to the zero mark, which indicates little or no variation between the variables. The little variability in the means of the subject matter, pedagogical expertise and the didactical expertise shows that these three constructs are closely associated with the formation of teacher identity. This is an indication that the sampled population attributed equal value to the three constructs towards the construction of their teacher identities.

The standard deviation (mean item standard deviation) was also computed and it gave a value of .04. The larger the standard deviation value, the greater the variability, and vice versa. Therefore, with a standard deviation of .04 from the computed means of the various constructs, it is confirmed that there is very little variation among the three constructs in the formation of teacher identity among this population.

## 4 Discussion

The finding of the study indicated a variance value of .0011, which is quite close to a variance of zero, indicates that the nursing student teachers accord a fairly balanced combination of subject matter expert, pedagogical expert and didactical expert towards the formation of their teacher identity. The value of the standard deviation (.04) also gives us a picture of how close the students combine these three concepts when forming their teacher identity. One could infer from this finding that they give equal importance to the three constructs during the process of teacher identity formation. Similar to the findings of this study, Beijaard et al. (2000a,b) also found that teachers saw themselves as a combination of subject matter experts, didactical expert and pedagogical experts. Nevertheless, in their study, the teachers gave more importance to subject matter expertise and didactical expertise than to pedagogical expertise. It is assumed that a balanced professional identity will lead to a wide range of competencies (Wilhelm, Brovelli, Rehm, & Kauertz, 2010). The finding suggests that a range of competencies assumes a balanced professional identity.

Obtaining a fairly balanced professional identity of the entire cohort could also mean that in spite of the professional teacher identity tensions faced by student nurse‐educators during their practicum period, they are still capable of adopting fairly balance professional teacher identities. This assertion is supported by the findings of a study conducted by Pillen, Den Brok, and Beijaard (2013) that suggested that the professional identity tensions tend to dissipate as student teachers navigate through the teacher training process, but do not completely disappear. The authors also suggested that the professional teacher identity of beginning teachers during the transition phase from final year to first year in‐practice is usually unstable.

The literature on teacher identity does not indicate to what extent the concepts of subject matter expert, pedagogical expert, and didactical expert, merge to form a balanced teacher identity. This is partly because there are other important contextual factors that play a role in the comprehensive formation of the teacher identity (Smit & Fritz, 2008). Nevertheless, a study conducted by Beijaard et al. (2000a,b) to explore the influence of the teaching context, the teaching experience and the biography of teacher revealed that contextual, experiential and biographical factors do not really have an influence on the perceptions of the teachers on their teacher identity. Therefore, the degree to which these concepts are combined to form the teacher identity may vary not only with the individual student nurse‐educator but also with the general institution based on various factors.

This research will contribute to forming an understanding of the roles of the subject content knowledge, pedagogical knowledge and didactic knowledge in particular as they affect teacher identity and teaching itself. The results of this study will contribute to the existing literature on teacher identity by adding new information regarding the cognitive aspect of student nurse‐educator's knowledge as it affects teacher identity. The suggestions emanating from the study could help nursing education programmes address teacher identity issues faced by student nurse‐educators. The study advocates the embedding of professional guidance and support in student nurse‐educator's teacher training.

## 5 Conclusion

The fundamental requirements for proficient teaching are based on a broad grounding in educational theories, knowledge on the subjects to be taught, of the skills to be developed, and of the curricular arrangements and materials that organize and embody that content. Also required is the knowledge of general and subject‐specific methods for teaching and for evaluating student learning; knowledge of students and human development; and the capacities and dispositions to employ such knowledge wisely in the interest of students. These aspects do not work in isolation, but they are all integral to the process of the learning‐teaching transaction. This implies that all three constructs are integral to the teacher identity. This study shows that student nurse‐educators are capable of associating subject matter expert, pedagogical expert and didactical expert in a balanced proportion during the formation of their teacher identity. Understanding how students from their professional teacher identity could offer educators the opportunity to balance the content of the curriculum for training student teachers to pay attention to building the three main aspects for a successful professional teacher identity formation. It could also provide pointers on how to induct student nurse‐educators into the real world of their work in a way that promotes a high level of professional practice and competencies.

### 5.1 Limitation and suggestions for future research

The first limitation of the study is related to the self‐report nature of the questionnaire. A self‐reporting questionnaire is inherently biased by the person's feelings at the time they fill out the questionnaire. The use of a questionnaire seemed to be the best way to extract information about the perceptions of student nurse‐educators, related to their teacher identity; however, at the same time, implies that no in‐depth information was gained. Also, the small sample of student nurse‐educators, within a specified research context might limit the extent of generalizability with regard to teacher identity of student nurse‐educators. Examination of the process through which student nurse‐educators constructed their teacher identity during teaching practice was limited to the three principal constructs of as subject matter expert, didactical expert, and pedagogical experts and did not take into consideration the influence of contextual factors identified in the framework.

It might also be beneficial to consider using a qualitative method to understand how student nurse‐educators combine the concepts of subject matter expert, pedagogical expert and didactical expert when forming their teacher identity. Using interview or focus group methods may also be useful to understand other factors and identity tensions that may be relevant in the formation process of teacher identity during the practicum phase of the training of student nurse‐educators.

## Conflict of interest

Non declared.
